# Analysis of compression damage pattern and strength influencing factors in graphite-tailing-filled soilbags

**DOI:** 10.1038/s41598-023-50349-0

**Published:** 2024-01-08

**Authors:** Jian Gao, Changbo Du, Zhan Xu, Fu Yi

**Affiliations:** 1 School of Civil Engineering, Liaoning Technical University, Fuxin, 123000 China; 2 School of Resources and Civil Engineering, Liaoning Institute of Science and Technology, Benxi, 117004 China; 3 Beijing Jingneng Geological Engineering Co., Ltd., Beijing, 102300 China

**Keywords:** Civil engineering, Mineralogy

## Abstract

To realize the resourceful use of soilbags filled with graphite tailings, their load-bearing and deformation characteristics must be fully understood. In this study, the following results were obtained by performing geometric testing of water-filled sealing bags and uniaxial compression tests of soilbags filled with graphite tailings. The volume of the soilbag expressed in rectangular form was approximately 0.773 times the actual volume. The types of compression damage to soilbags can be defined as surface damage and overall damage. The surface damage load increases with decreasing filling density and decreases with decreasing soilbag size. Moreover, the higher the tensile capacity of the soilbag material and the lower the friction between the soilbags, the greater the surface damage load. The overall damage load increased with an increase in the tensile strength of the soilbag material and decreased with an increase in the degree of filling; the overall damage load was greater for large-sized soilbags at high degrees of filling. Thus, the existing theoretical calculation method cannot accurately calculate the damage load of soilbags filled with graphite tailings, and the test results deviate from the theoretical calculation results, with the latter showing an increasing damage load with a decreasing filling degree.

## Introduction

Graphite is commonly used in the manufacture of graphite electrodes, high-temperature clean energy sources, high-performance lithium-ion batteries, and carbon-fiber materials^1^. Approximately 70% of the total graphite and 100% of the total uncoated spherical graphite worldwide are mined in China^2,3^. However, the amount of graphite tailings produced in China has increased rapidly with the increase in graphite production, causing serious environmental problems^4,5^. The main applications of graphite tailings include building materials^6–10^ road subbases, ceramic tiles^11^, and decoloring agents^12^, among others.

Soilbags are advantageous for treating solid waste^13,14^. Currently, they are widely used in slope protection^15,16^, scour control^17^, riverbank protection^18^, waste treatment^19^, foundation treatment^20–22^, retaining walls^23–25^, road engineering^26,27^, and tailing dams^28–30^. To realize the resourceful use of soilbags filled with graphite tailings, their load-bearing and deformation characteristics must be fully understood. Several researchers have investigated the performances of soilbags. Liu et al.^26^ and Wang et al.^31^ demonstrated a good vibration damping effect of soilbags through experiments and the discrete element method (DEM). Matsushima et al.^32^ proposed a method for the inclined stacking of earthbags, which showed greater shear strength when stacked inclined than horizontally. Fan et al.^25^ designed a shear test using multiple layers of vertically stacked soilbags and demonstrated that the interlayer frictional resistance of the soilbags was related to the shape of the sliding surface. Regarding the compressive properties of soilbags, Matsuoka and Liu^33^ proposed a simplified analytical scheme for the compressive strength of earthbags under plane-strain conditions by introducing an apparent cohesive force based on the assumption of uniformly distributed tensile forces on the soilbag and a frictionless soilbag interface. Bai et al.^34^ proposed the principle of a three-dimensional stress state analysis of soilbag reinforcement, and derived an expression for the ultimate strength under three-dimensional complex stresses based on the Mohr–Coulomb damage criterion. Liu et al.^35^developed a two-dimensional strength formulation for soilbags under inclined loading, which was further verified via DEM simulations. Tantono^36^ simplified the stress state assumption by setting the stress ratio to a constant value at different locations. The aforementioned analytical scheme assumes a constant volume of encapsulated soil during compression, and can only be applied to specific encapsulated soils under specific soilbag geometry conditions. Cheng et al.^37^ and Cheng et al.^38^ suggested that the soil wrapped by geotextiles may be a fully elastic–plastic solid under triaxial conditions, assuming that the fill soil is governed by the Mohr–Coulomb yield criterion, and its expansion is related to the principal stress ratio. They further proposed an analytical solution for soilbags based on simulations. Shen et al.^39^ proposed a unified stress–strain model for geotextile-wrapped soils, and applied it to understand the strength and deformation characteristics of soilbags under two-dimensional biaxial loading. Considering the relationship between the tensile force and vertical strain of an earthbag, Jia et al.^40^ derived formulae for predicting the strength of an earthbag and verified the results through DEM simulation laboratory tests. Liu et al.^41^ conducted a series of unconfined compression tests under monotonic and cyclic loading, and proposed empirical equations to describe the changes in the cumulative vertical strain and modulus of elasticity of stacked earthbags under different vertical stress and cyclic load ratios, respectively.

The aforementioned studies are not sufficiently accurate to describe the characteristics of the soilbag damage pattern, and the mechanisms of the influence of the filling degree, soilbag size, and other factors on the strength have not been sufficiently analyzed. Accordingly, in this study, a method for calculating the height of soilbags with different degrees of filling was developed through a water injection test of sealed bags. Subsequently, single- and multi-layer unconfined compression tests were performed with different soilbag materials, soilbag sizes, and degrees of graphite-tailing filling. Overall, the damage load and deformation characteristics of graphite tailings filled with soilbags were obtained under different damage modes, the effects of different factors on the damage load were analyzed, and the relationship between the test results and theoretical calculations was derived. The results of this study are thus valuable for the application of soilbags filled with graphite tailings and for improving force analysis models.

## Experimental program

### Experimental materials

#### Geotextiles

**Figure 1:**
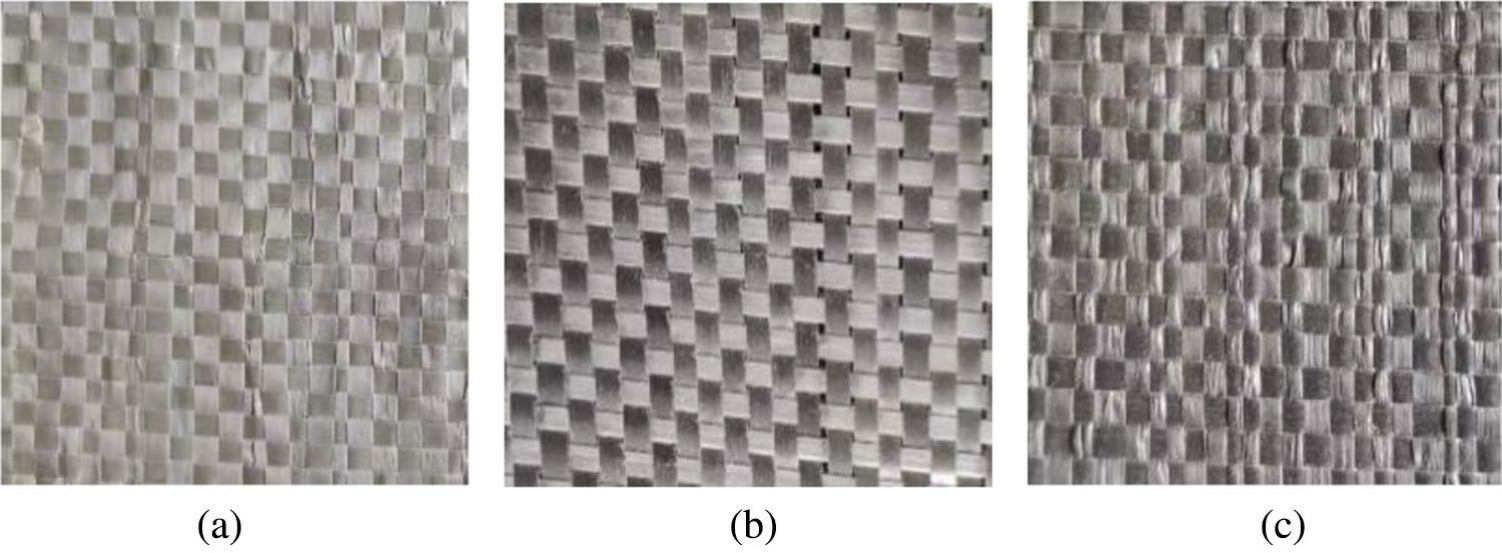
Geotextiles used for the experiment: (**a**) 80 g/m^2^, (**b**) 200 g/m^2^, and (**c**) 300 g/m^2^.

The geotextile was a polypropylene woven geotextile produced by an engineering material company in Shandong, China, as shown in Fig. 1. According to ASTM D 5261 and ASTM D 4595, the mass per unit area and tensile tests were conducted for the three types of geosynthetics, and the test results are specified in Table 1.

**Table 1:** Basic properties of the geotextiles.

Physical index	Unit	Test results			Test method
Mass per unit area	g/m^2^	84	202	306	ASTM D 5261
Warp strength	KN/m	18	41	62	ASTM D 4595
Weft strength	KN/m	12	30	42	ASTM D 4595
Longitudinal elongation	/%	13	18	16	ASTM D 4595
Latitudinal elongation	/%	9	15	12	ASTM D 4595

#### Properties of graphite tailings

**Figure 2:**
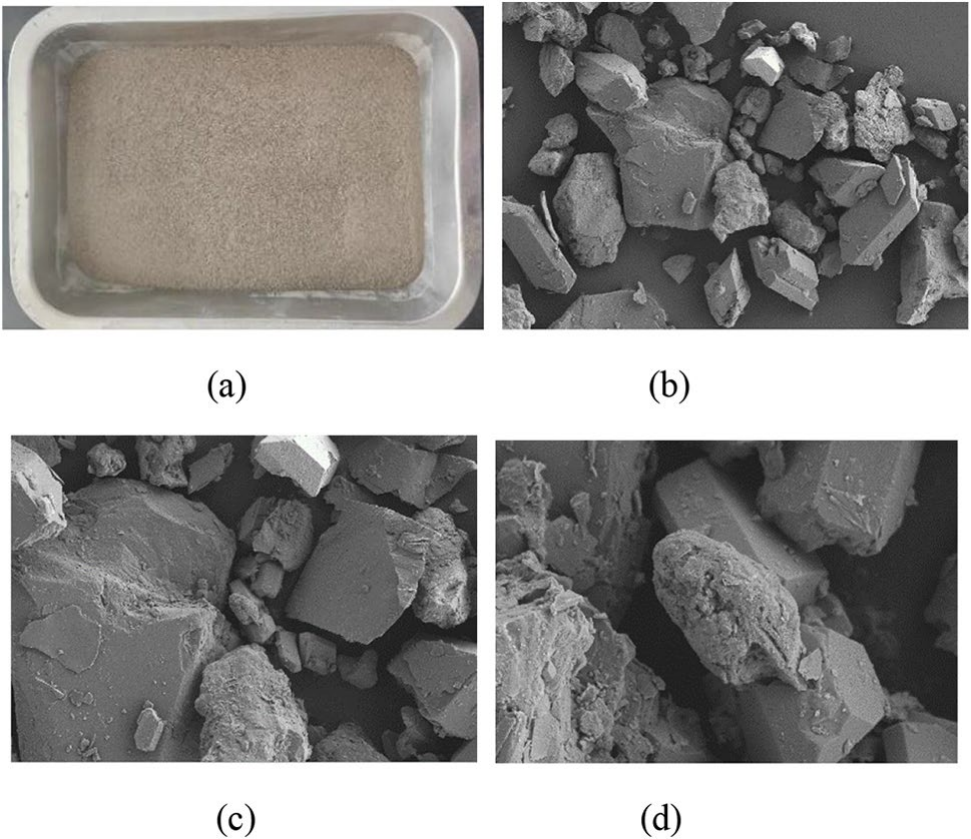
Graphite tailings in actual and electron microscopic conditions: (**a**) actual, (**b**) 500 × , (**c**) 1000 × and (**d**) 5000 × .

**Figure 3:**
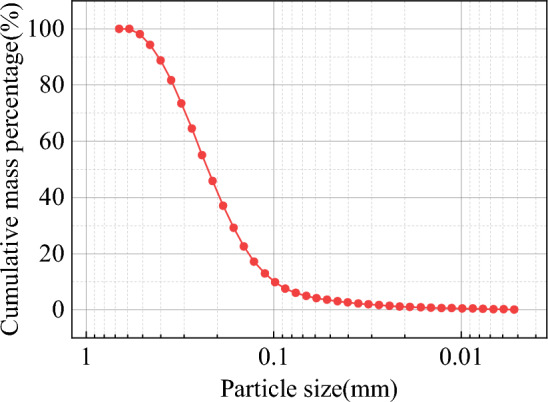
Gradation curves of the graphite tailings.

Graphite tailings were taken from a graphite tailings depot in Luobei County, Heilongjiang Province, and the actual situation and the graphite tailings scanned via SEM are displayed in Fig. 2. The basic physical properties of the graphite tailings (Table 2) were determined according to the geotechnical test method standard (GB/T 50123-2019)^42^. The particle size of the graphite tailings, whose graduation curves are shown in Fig. 3, was determined using a Betterize 2600 laser particle size analyzer.

**Table 2:** Basic physical properties of the graphite tailings.

Material	Maximum dry density (g/cm^3^)	Minimum dry density(g/cm^3^)	Maximum porosity ratio	Minimum porosity ratio	Specific gravity (g/cm^3^)	Bulk density (g/cm^3^)	Cohesive (kPa)	Friction angle(°)
Graphite tailings	1.41	1.94	0.95	0.41	2.75	1.41–1.62	0	27.3–32.9

**Figure 4:**
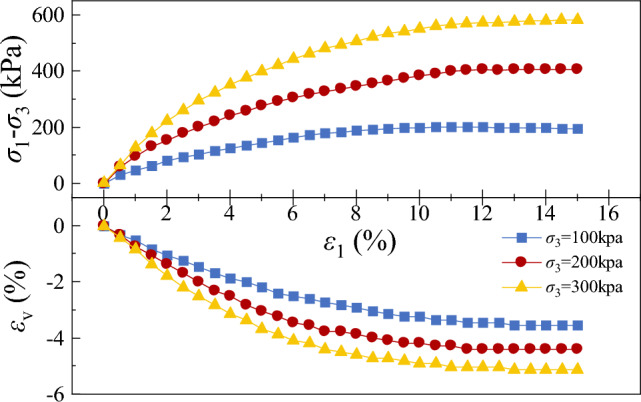
Stress–strain curve and volume strain-axial strain curve.

**Figure 5:**
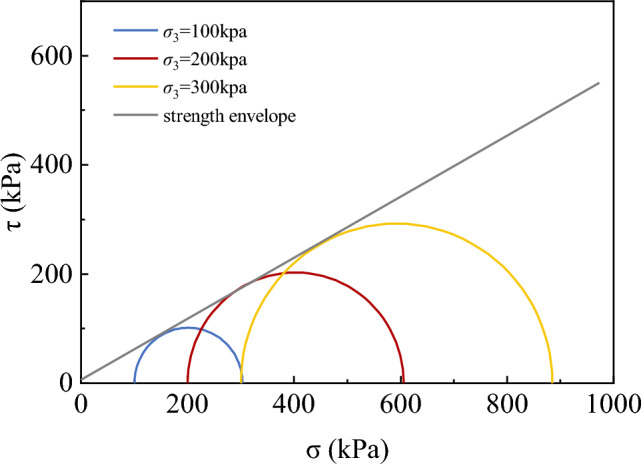
Shear strength envelope of triaxial test.

To understand the shear strength and volume change characteristics of graphite tailings, the STD-10 geotechnical triaxial test system produced by Xi’an Lichuang Material Testing Technology Co., Ltd. was adopted to carry out consolidation and drainage shear tests on saturated specimens of graphite tailings. The dry density of the specimens was 1.7 g/cm^3^ and the deformation control method was used with the loading rate of 0.08 mm/min. Compression tests were performed at confining pressures of 100, 200, and 300 kPa, stopping the test when the strain reached 15%. Figures 4 and 5 present the results of triaxial shear tests on the graphite tailings.

From Fig. 4, it can be observed that the deviatoric stress increased with the increase of strain, and until the end of shear, the deviatoric stress did not show any obvious peak point. The volumetric strain increased with an increase in the axial strain, indicating that the graphite tailings decreased in volume during the shear process. According to the test results, the strength envelope of the graphite tailings was organized as shown in Fig. 5. According to the Mohr–Coulomb strength theory, the cohesive force and angle of internal friction of the graphite tailings were determined to be 29.22° and 5.93 kPa, respectively.

#### Shear properties between materials used

**Figure 6:**
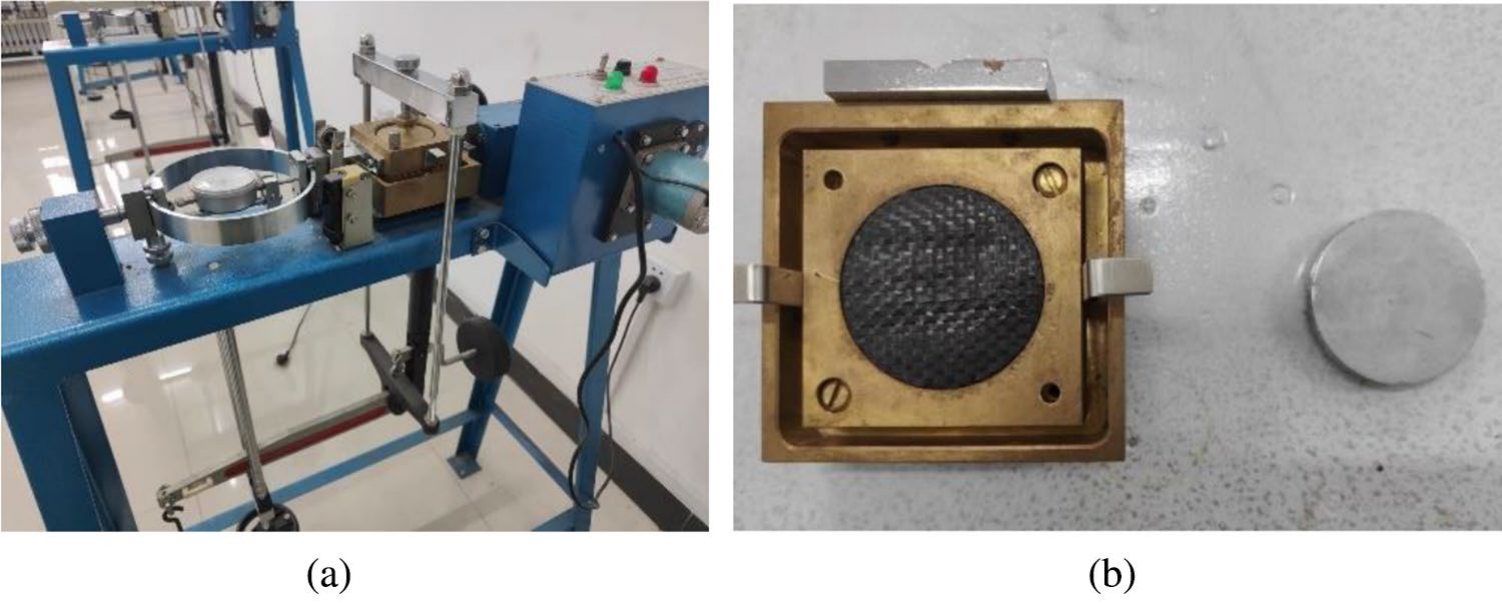
Test equipment and shear interface: (**a**) ZJ-2 strain-controlled straight shear equipment, (**b**) geosynthetics and metal plate interface.

To accurately grasp the interfacial shear characteristics between the materials used, the interfacial shear characteristics of geosynthetics and geosynthetics, geosynthetics and tailings sand, and geosynthetics and metal plates were tested using ZJ-2 strain-controlled straight shear equipment produced by Nanjing Ningxi Company and with reference to the standard of the geotechnical test method (GB/T 50123-2019)^42^. The test equipment and shear interface are shown in Fig. 6. The results of these tests are presented in Table 3.

**Table 3:** Shear properties between materials used.

Geotextile mass per unit area (g/m^2^)	80		200		300	
Shear strength index	Cohesive (kpa)	Friction angle (°)	Cohesive (kpa)	Friction angle (°)	Cohesive (kpa)	Friction angle (°)
Geosynthetics and metal plate	0	11.61	0	12.21	0	13.84
Geosynthetics and geosynthetics	0	16.79	0	18.22	0	22.51
Geosynthetics and graphite tailing	0	23.48	1.36	26.23	2.27	27.02

### Experimental process

To study the geo-bag geometry before compression, water was used to fill different sizes of polyethylene sealed bags, and a 0.3 m × 0.3 m transparent acrylic sheet was placed flat on the top to measure the height of the bag and the contact size with the acrylic sheet.

The soilbags used in previous studies were mainly hexahedral^43^ and flat rectangular^26^ in shape. In this study, a test was conducted using rectangular soilbags prepared according to the size of the press-bearing plate to achieve side lengths of 0.2 m × 0.2 m and 0.25 m × 0.25 m. During the fabrication process, the geotextile was shaped into a rectangle with a length twice that of the side plus 5 cm and a width of the side plus 10 cm and folded in half along its length. The edges were sewn into a double J shape using a sealing machine^44^ with openings in the width direction for subsequent filling with graphite tailings.

The dried graphite tailings were placed into well-made soilbags that continuously vibrated during the sand-filling process until they were full. The masses of the 25-cm and 20-cm soilbags when filled were 4450 g and 2250 g, respectively. The dry density of graphite tailings in the soilbag was calculated to be approximately 1.48 g/cm^3^, with the pore ratio of 0.86 and relative density of 0.17, which is considered a loose state. The soilbags were filled with graphite tailings to 100%, 80%, and 60% of the maximum fill mass.

To analyze the compression damage characteristics of the soilbags, the specimens were prepared according to Table 4 for the uniaxial compression test.

**Table 4:** Compression test specimens of multi-layer soilbags.

Soilbag size (cm)	Layers	Geotextile mass per unit area (g/ m^2^)	Fill rate (%)
20	1, 2, 3, 4, 5, 6	80	100

To further analyze the effect of single versus multi-layer, geotextile unit area mass, degree of filling, and soilbag size on the compressive properties of the soilbags, specimens were prepared according to Table 5 and subjected to uniaxial compression tests.

**Table 5:** Specimen information.

Soilbag size (cm)	Layers	Geotextile mass per unit area (g/ m^2^)	Fill rate (%)		
20	3	80	60	80	100
	1		100		
	3	200	60	80	100
	1		100		
	3	300	60	80	100
	1		100		
25	3	80	60	80	100
	1		100		
	3	200	60	80	100
	1		100		
	3	300	60	80	100
	1		100		

**Figure 7:**
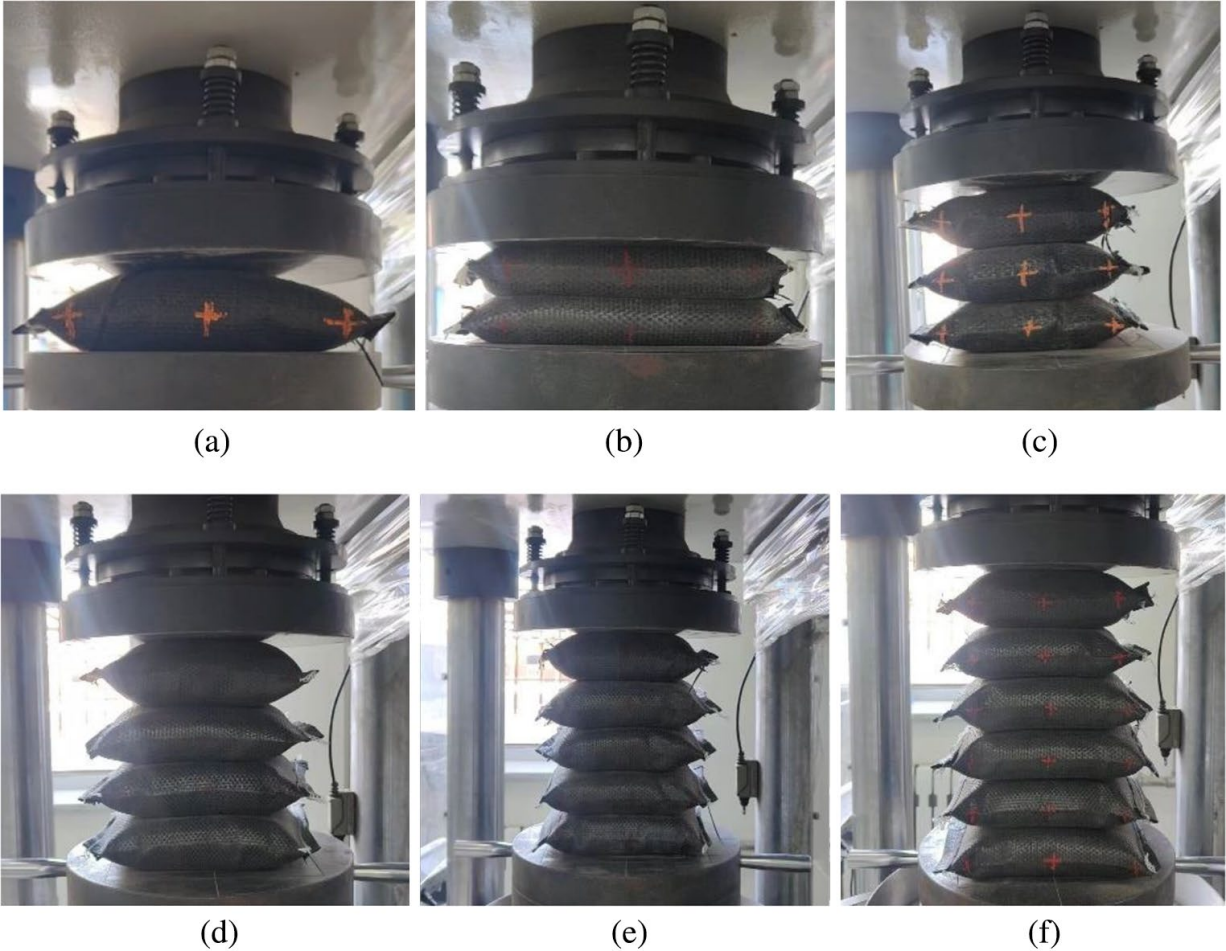
Uniaxial compression test of soilbags: (**a**) single layer, (**b**) two layers, (**c**) three layers, (**d**) four layers, (**e**) five layers, and (**f**) six layers.

A TYE-3000KE-type microcomputer-controlled automatic pressure tester was used as loading equipment. Before placing the soilbag, clean the bearing plate and apply a small amount of grease to reduce friction on the plate. The loading rate and maximum loading were set to 5 kN/s and 2800 kN, respectively, and the soilbag was pressurized to 10 kN before loading. To reduce errors, three parallel tests were conducted on each group of specimens and the arithmetic mean of the damage load was used as the test result. In the three tests, if the test result exceeded ± 10% of the mean value, that specimen result was rejected and the mean value of the remaining specimen results was used as the test result. If the results of the remaining specimens still exceeded ± 10% of the mean, the test was invalidated and all three specimens were prepared again for testing. Figure 7 shows the loading of soilbags.

## Results and analysis

### Geometric characteristics of the soilbags before loading

The study of the initial compression of soilbags is the basis for analyzing their compression deformation, and accurately determining the initial morphology of soilbags with different filling degrees helps calculate the size of the soilbags after stacking. The maximum filling volume of a rectangular soilbag belongs to the “paper bag problem”^45^, which can be calculated using Eq. (1), where a and b are the long and short sides of the bag, respectively.

1 $$V = a^{3} \left[ {\frac{b}{\pi a} - 0.142\left( {1 - 10^{ - b/a} } \right)} \right].$$

**Figure 8:**
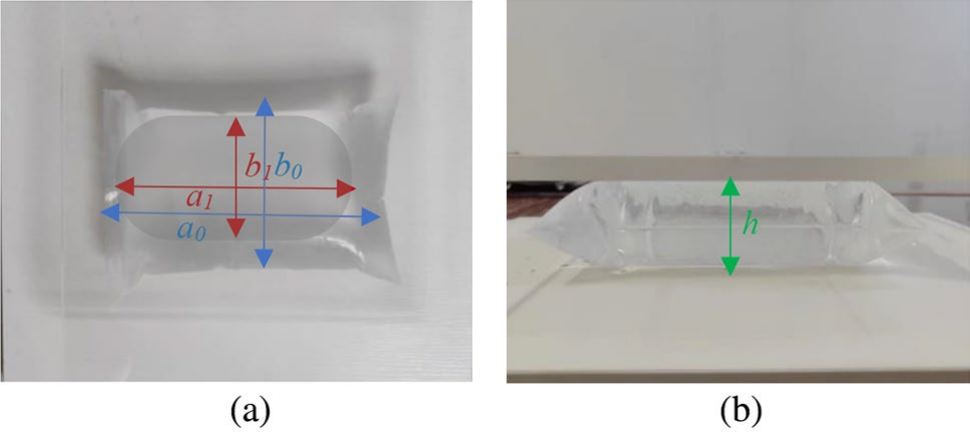
Shape of the water-tight bag: (**a**) top view and (**b**) side view.

A water-injection test was performed on a sealed polyethylene bag to determine the geometric properties of the soil bag before loading. The test indices and results are presented in Fig. 8 and Table 6, respectively.

**Table 6:** Geometry of the filled sealed bag.

Bag size		Geometric indicators					Mass (g)	Calculated volume (cm^3^)
a (cm)	b (cm)	h (cm)	a_0_ (cm)	a_1_ (cm)	b_0_ (cm)	b_1_ (cm)		
18	17	5.51	13.73	10.86	12.77	9.88	998.2	1019.129
21	18	6.07	16.21	13.23	13.20	10.23	1345	1394.409
23	22	7.9	15.82	11.81	16.94	12.64	2098.3	2167.74
28	27	9.25	20.52	16.38	19.83	14.98	3865.2	3959.006
31	30	11.24	21.21	15.43	22.21	16.51	5320.1	5401.92
39	30	12	29.64	23.53	20.60	14.53	7450	7534.199

As shown in Table 6, the volume of the water-filled Ziplock bag is essentially the same as that calculated using Eq. (1). The shape of the bag side was somewhere between a straight line and a semicircle. To quickly calculate the pressurized area, the soilbag can be simplified as a cuboid. Assuming that the length, width, and height of the flat bag body are *a*, *b*, and *h*, respectively, the simplified volume *V*_1_ can be calculated using Eq. (2).

2 $$V_{1} = abh - (a + b)h^{2} + h^{3} .$$

**Figure 9:**
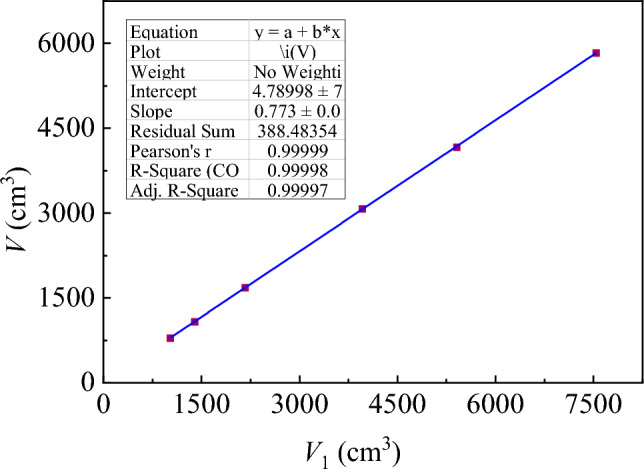
Volume by cuboid vs. maximum volume.

The volumes *V* and *V*_1_ were calculated by substituting the relevant parameters from Table 6 into Eqs. (1) and (2), respectively: Fig. 9 shows the relationship between *V* and *V*_1_.

**Figure 10:**
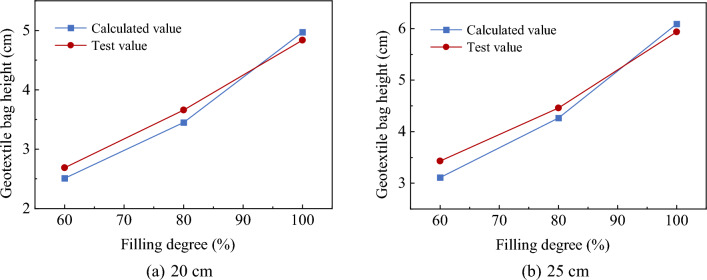
Comparison of theoretically calculated and measured heights.

As shown in Fig. 9, the cuboid-calculated volume *V*_1_ is approximately 0.773 times the maximum volume *V*. Considering this relationship besides Eqs. (2), the soilbag height can be determined for different fill levels. Based on the above analysis, the relationship between the heights of the experimental 25- and 20-cm soilbags at different filling degrees and the calculated heights is presented in Fig. 10.

The above comparison shows that the deviation of the calculated geocell height from the measured value is within 10%, which verifies the feasibility of the method for estimating the height of geocells with different filling degrees.

### Compression damage of soilbags of different layers

**Figure 11:**
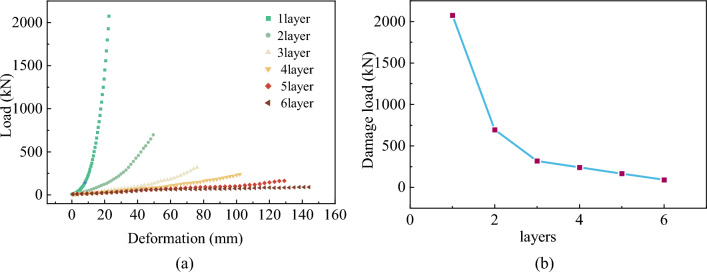
Compression test results of soilbags with different number of layers: (**a**) Load-deformation relationship, (**b**) Ultimate load-layer relationship.

The test results for the compression damage of soilbags with different numbers of layers are shown in Fig. 11.

From Fig. 11a, it can be seen that the load increases nonlinearly with the increase in displacement, and the growth rate is slow in the pre-loading period. With the gradual densification of the tailings in the soilbag, the growth rate of the load gradually accelerates and tends to stabilize. The growth rate of the load decreases with an increase in the number of layers. As can be seen from Fig. 11b, the compressive capacity of single-layer soilbag is the strongest, and the compressive capacity of soilbag decreases as the number of layers increases. The destructive load of the soilbag gradually stabilized after more than three layers.

**Figure 12:**
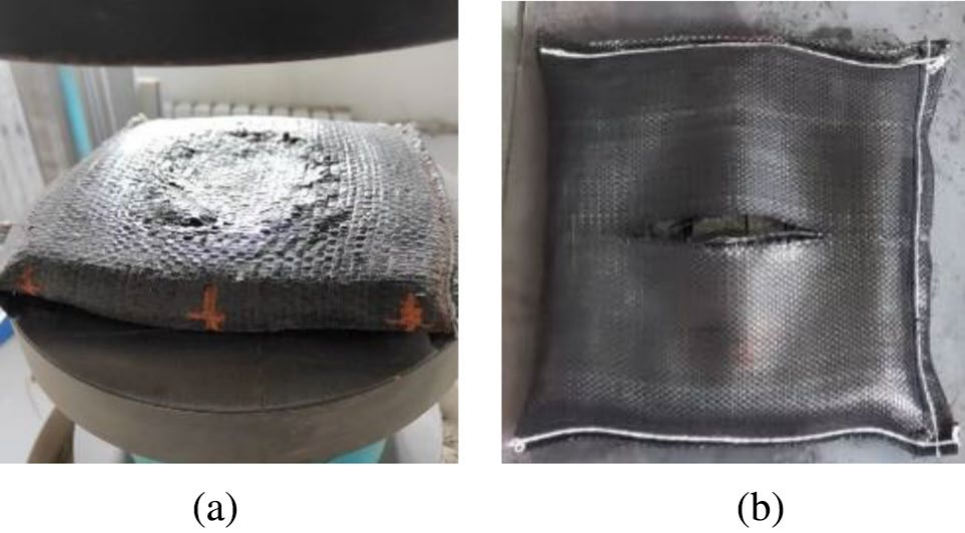
Surface damage of soilbags: (**a**) surface damage of a single soilbag, (**b**) surface damage of the intermediate layer of a multi-layer soilbag.

During the loading process, the capacity of the soilbag decreases with the height, and at the same time, the volume of the graphite tailings decreases with the increase in compactness. When the volume of the graphite tailings decreases less than the volume of the soilbag capacity decreases, a tensile force will be generated within the geotextile.With the growing tension within the geotextile, the surface of the soilbag first reaches the damage tension to crack, as shown in Fig. 12.The vertical load to which the soilbag is subjected when surface cracking begins to occur is defined as the surface damage load. The upper and lower contact surfaces of the single-layer soilbags were in contact with the bearing plate, while multi-layer soilbags have contact between soilbags in addition to contact with the bearing plate. The surface damage of the single-layer soilbag was ring-shaped, and the surface damage of the multi-layer soilbag was cracking damage in the middle of the soilbag along a certain direction.

**Figure 13:**
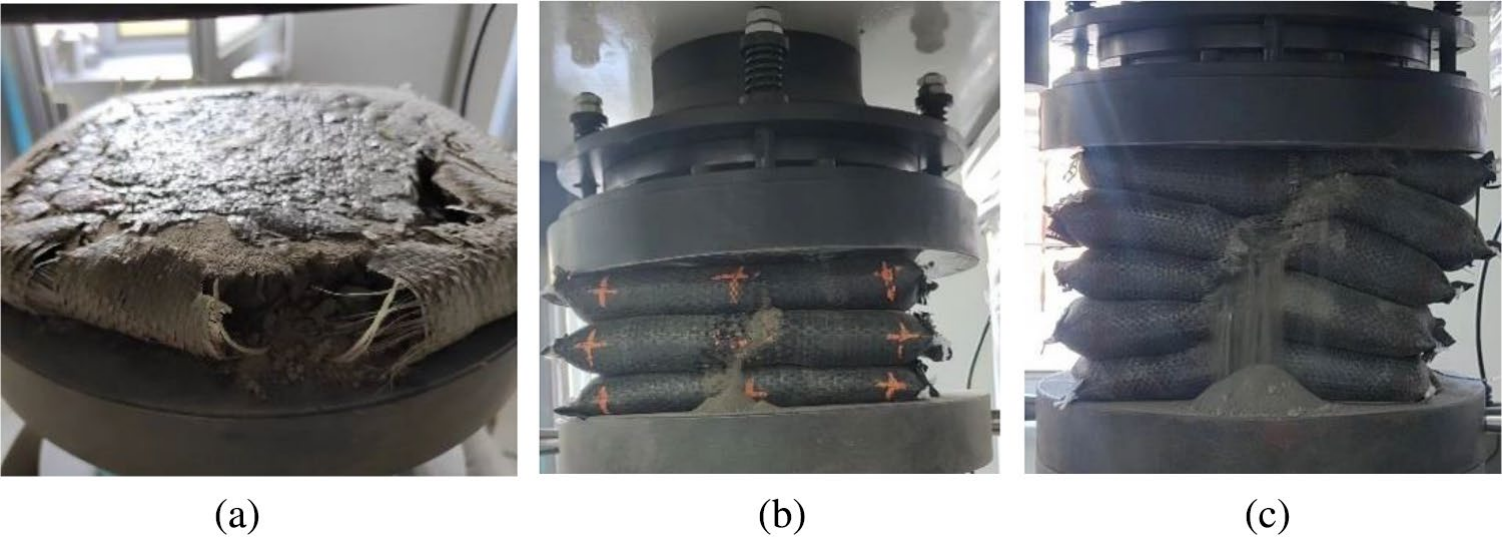
Overall damage soilbag damage: (**a**) single layer soilbag, (**b**) lateral damage of intermediate layer, (**c**) overall shear damage.

When surface damage occurs, the soilbag can withstand the load. As the load increased, the surface damage continued to develop until there was tensile damage on one side of the soilbag, and for multi-layer soilbags, the middle layer was the first to experience lateral damage. After lateral damage to the soilbag occurred, sand began to seep out, and overall shear damage increased gradually as the number of layers increased, as shown in Fig. 13. The vertical load at which lateral cracking occurred in the soilbag was defined as the overall damage load.

### Analysis of factors affecting compressive properties of soilbags filled with graphite tailings

To analyze the effect of the number of soilbag layers, geotextile material, degree of filling, and soilbag size on the compressive properties of the soilbags, uniaxial compression tests were performed according to the specimen scheme in Table 5. Because the damage load of soilbags gradually tends to stabilize when the number of soilbag layers exceeds three, three-layer soilbags were used as a representative of multi-layer soilbags for comparison with single-layer soilbags.

#### Damage loads and deformation of soilbags

**Figure 14:**
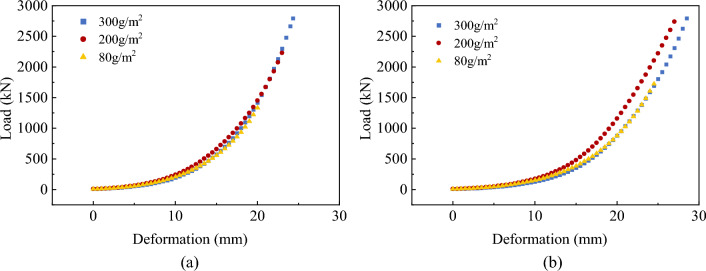
Compression test results of single-layer soilbags: (**a**) 20 cm and (**b**) 25 cm.

The load-deformation curve for the compression damage of a single-layer soilbag is displayed in Fig. 14.

Figure 14 shows that the load increases with an increase in deformation. When the deformation was 0–10 mm, the load increased slowly; when the deformation was 10–20 mm, the growth rate of the load increased; and when the deformation was greater than 20 mm, the load and deformation experienced approximately linear growth. For 20-cm soilbags with masses per unit area of 80, 200, and 300 g/m^2^, the surface damage loads were 44.640, 124.103, and 157.538 kN, and the corresponding deformations were 4.0992, 7.264, and 9.182 mm, respectively. For 25-cm soilbags of different materials, the surface damage loads were 51.619, 137.005, and 227.805 kN, and the corresponding deformations were 5.225, 8.877, and 12.002 mm, respectively. The above deformation results indicate that the deformation was approximately 14.6% of the height before compression when surface damage occurred. At 20 cm, the overall destructive loads of soilbags with masses per unit area of 80 and 200 g/m^2^ were 1363.437 and 2150.3822 kN, respectively, and the corresponding deformations were 20.129 and 22.755 mm, respectively. At 25 cm, the overall destructive load of the 80-g/m^2^ soilbag was 1747.575kN, and the corresponding deformation was 24.596 mm. When the soilbag underwent overall damage, the deformation was approximately 45.1% of that before it was pressurized. At 25 cm, 200- and 300-g/m^2^ soilbags can resist a load of 2800 kN without lateral tearing. Conversely, at 20 cm, 300-g/m^2^ soilbags can resist the same load without lateral tearing.

**Figure 15:**
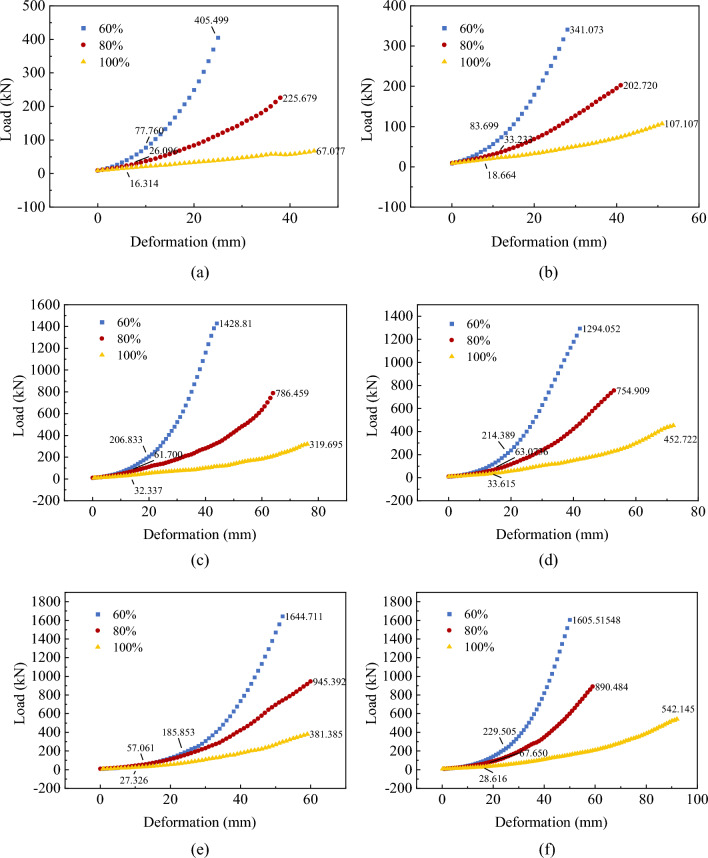
Load-deformation curves of three-layer soilbags: (**a**) 80 g/m^2^, 20 cm, (**b**) 80 g/m^2^, 25 cm, (**c**) 200 g/m^2^, 20 cm, (**d**) 200 g/m^2^, 25 cm, (**e**) 300 g/m^2^, 20 cm, and (**f**) 300 g/m^2^, 25 cm.

The compressive load-deformation curves of the three-layer soilbags for each degree of filling are shown in Fig. 15, where the surface and overall damage loads are labeled.

Figure 15 shows that the load increased with increasing deformation, and the rate of increase increased with decreasing degree of filling. There was no obvious inflection point in the load–displacement curve of the soilbag with a 100% filling degree. For the 80%- and 60%-filled soilbags, the inflection point of the load occurred near 1/2 and 1/3 of the overall destructive deformation, and the growth rate increased further and tended to be linear after the inflection point. The corresponding compression deformations of soilbags with filling degrees of 100%, 80%, and 60% were 6.476%, 10.778%, and 20.245% of the initial height in the case of surface damage and 39.766%, 43.674%, and 45.43% of the initial height in the case of overall damage. Thus, the smaller the degree of filling, the greater is the deformation at damage. As shown in Fig. 15, the surface and overall damage loads of the soilbag filled with graphite tailings are related to the soilbag material, degree of filling, and size of the soilbag.

#### Effect of number of soilbag layers on damage load of soilbags

**Figure 16:**
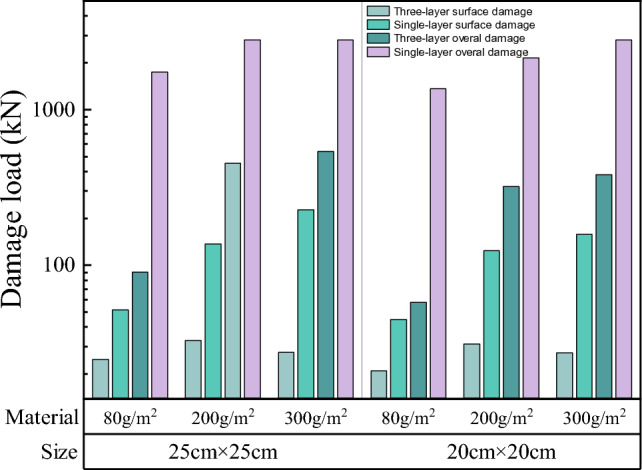
Comparison of the damage loads of single- and multi-layer graphite-tailing-filled soilbags.

A comparison of the damage loads of single- and three-layer soilbags with 100% fill is shown in Fig. 16.

As can be seen in Fig. 16, the overall surface damage loads of the three-layer soilbags were lower than those of the single-layer soilbags. The surface damage loads of the single-layer soilbags with mass units of 80, 200, and 300 g/m^2^ were 2.107, 4.086, and 7.017 times higher than those of the three-layer soilbag, respectively, whereas the overall damage load of the single-layer soilbag with a mass per unit area of 80 g/m^2^ was 18.321 times higher than that of the three-layer soilbag. Single-layer soilbags with unit-area masses of 200 and 300 g/m^2^ failed to measure the overall damage loads.

Because the interlayer friction of the soilbags is greater than the friction between the bag and bearing plate, the surface damage load of multi-layer soilbags is lower than that of single-layer soilbags.After the surface was damaged, the soilbag was in a lateral-bound state. Due to the low height of the single-layer soilbag, the lateral deformation capacity was weak. Under the protection of the pressure plate, the internal soil was constantly compressed densely, which further led to a lack of lateral tensile force. The soilbags with masses per unit area of 200 g/m^2^ and 300 g/m^2^ did not rupture, even at 2800 kN. The comprehensive test results and practical application conditions indicate that the multi-layer soilbags are closer to the actual force situation. Therefore, the subsequent discussion is based on multi-layer soilbags.

#### Effect of different geotextile materials on the damage load of soilbags

**Figure 17:**
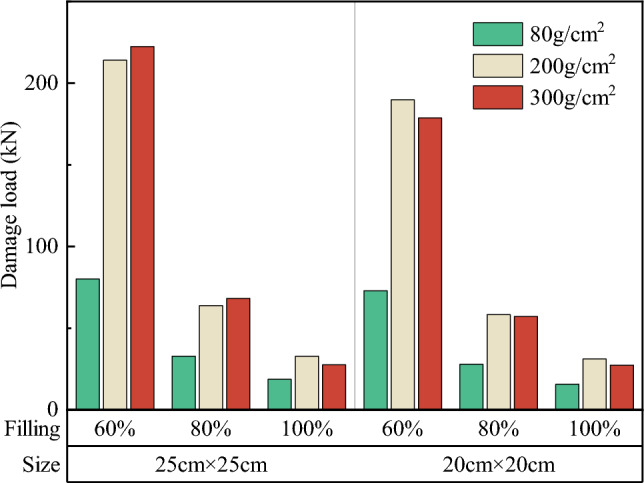
Comparison of the surface damage loads of soilbags with different materials.

**Figure 18:**
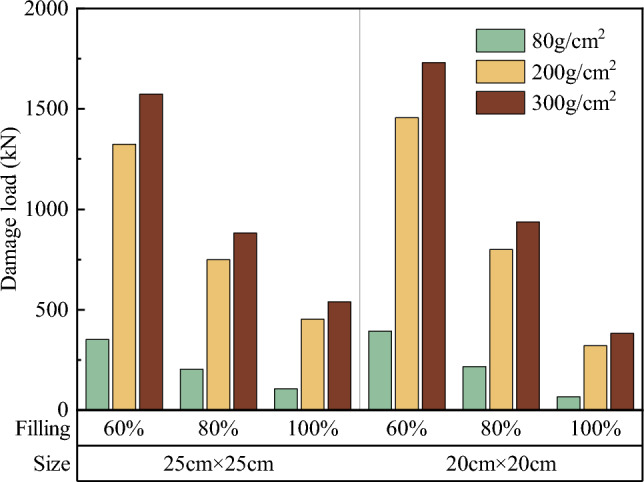
Comparison of the overall damage loads of soilbags with different materials.

Comparisons of the surface and overall damage loads of soilbags with different materials at each filling degree are shown in Figs. 17 and 18, respectively.

As shown in Fig. 17, the surface damage load of a 20-cm soilbag increases with increasing mass per unit area of the soilbag at 60% and 80% fill levels. Among the 25-cm soilbag (100% filled) and 20-cm soilbags at each fill level, the 200-g/m2 soilbags had the highest surface damage loads. As shown in Fig. 18, the overall damage load increased with increasing mass per unit area of the soilbag for each filling degree of the soilbag. Figures 17 and 18 show that the mass per unit area of the soilbag has a pronounced effect on the overall damage load and little effect on the surface damage load, and the surface damage strengths of the 200-g and 300-g soilbags are similar.

Previous studies^33^ have shown that one of the main sources of strength in soilbags is the tensile strength of the soilbag material. The higher the tensile strength, the stronger the compressive strength of the soilbag. However, for multi-layer soilbags, the role of friction between layers should not be ignored, as the friction between layers accelerates the tensile damage of the soilbags. From the above results, it can be seen that although the tensile strength of the 300-g/m^2^ material is greater, the surface damage load of the soilbag under the action of friction is close to that of the 200-g/m^2^ material.

#### Effect of different filling degrees on the damage load of soilbags

**Figure 19:**
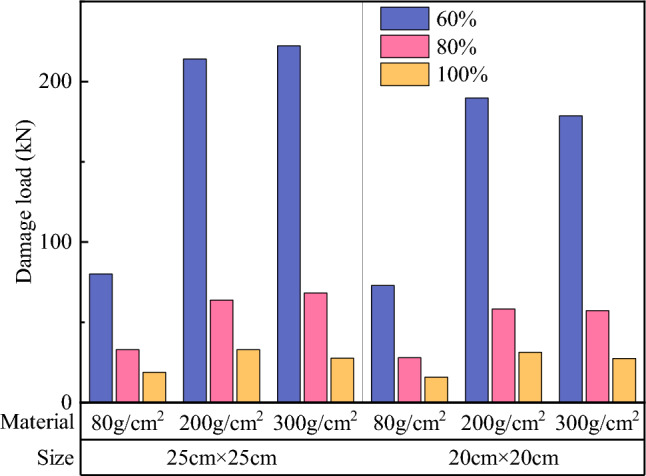
Comparison of the surface damage loads at different filling degrees.

**Figure 20:**
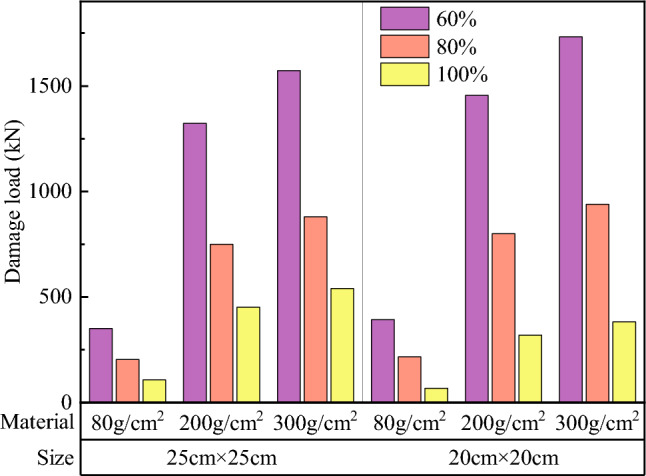
Comparison of the overall destructive loads at different filling degrees.

Comparisons of the surface and overall damage loads of the graphite-tailing-filled soilbags at different degrees of filling are shown in Figs. 19 and 20, respectively.

Figures 19 and 20 show that the surface and overall damage loads of the graphite-tailing-filled soilbags of the same material and size decreased as the degree of filling increased. The surface damage load of the 100%filled soilbag is approximately 17.4% of the 60%filled soilbag, and that of the 80%-filled soilbag is approximately 33.7% of the 60%filled soilbag. In contrast, the overall damage load of the 100%filled soilbag was 24.8% of the 60%filled soilbag, and that of the 80%filled soilbag was 53.1% of the 60%filled soilbag.

The main reason why a low-filling-degree soilbag is stronger than a high-filling-degree soilbag is that the height-to-width ratio of a low-filling-degree soilbag is smaller; thus, the cohesion generated by the soilbag wrapping is greater. Moreover, the compaction of the internal tailings after compression differed depending on the degree of the specimen filling. At a low filling degree, before reaching the overall damage, the internal graphite tailing compaction was high, internal friction angle was large, lateral tension force was weakened, and overall bad load was greater.

#### Effect of soilbag size on soilbag strength

**Figure 21:**
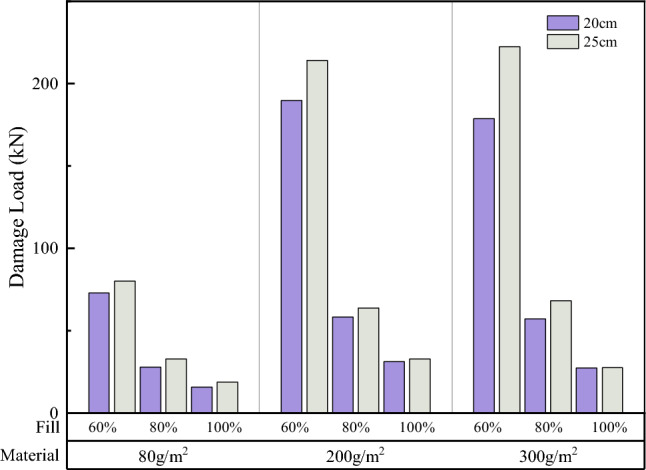
Comparison of the surface damage loads for different soilbag sizes.

**Figure 22:**
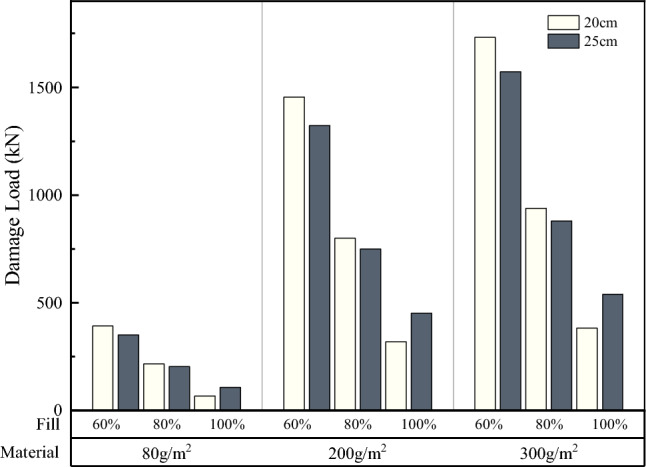
Comparison of overall damage loads for different soilbag sizes.

Figures 21 and 22 show the surface and overall damage loads for different soilbag sizes.

Figure 21 shows that the surface damage load at 20 cm was less than that at 25 cm for soilbags of the same material at all degrees of filling. The surface damage loads of 20-cm soilbags were approximately 86.7%, 86.8%, and 92.8% of those of the 25-cm soilbags at 60%, 80%, and 100% fill levels. As shown in Fig. 22, the overall damage loads were greater for 20-cm soilbags with 60% and 80% fill for the same material, whereas the overall loads of 25-cm soilbags with 60%, 80%, and 100% fill were approximately 90.4%, 93.7%, and 147.5% of that of the 20-cm soilbags.

Under the same degree of filling and soilbag material conditions, the surface damage strength of the small soilbag was higher than that of the large soilbag, indicating that the small soilbag interlayer friction was less than that of the large soilbag. This is due to the fact that small-sized soilbags can be easily compacted, and the tendency of the relative movement between the layers is less than that of the large-sized soilbag, and this effect becomes more pronounced as the fill level decreases. At 100% filling degree, the overall damage load is at a lower level, and the graphite tailings inside the bag are insufficiently compacted and remain in a loose state; therefore, the overall damage load of the small-sized geosynthetic bag is less than that of the large geosynthetic bag. At 60% and 80% fill, the internal compaction effect of small soilbags was greater than that of large soilbags, resulting in an increase in their internal friction angle; therefore, the overall damage load of small soilbags was greater than that of large soilbags.

### Comparative analysis of test results and theoretical calculations

#### Theory of strength calculation of soilbags

**Figure 23:**
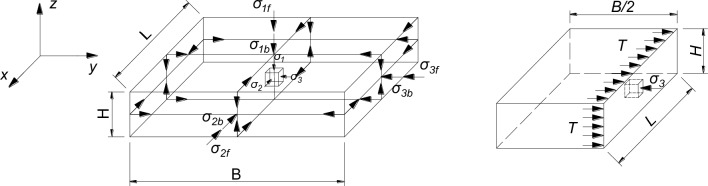
Three-dimensional model of the stress state of the soil inside the soil bag.

Currently, the analysis of soilbag strength is primarily based on a two-dimensional force analysis model proposed by Matsuoka^33^. The Matsuoka force analysis model was extended to a three-dimensional model, and the force analysis diagram is shown in Fig. 23. *σ*_1b_, *σ*_2b_ and *σ*_3b_ denote the stress increment caused by the bag tension. *σ*_1f_, *σ*_2f_ and *σ*_3f_ denote the magnitude of the principal stress at the destruction of the soilbag.* T* denotes the destructive force of the geosynthetics. The model assumes that the direction of the principal stresses is perpendicular to the surface of the soilbag, where the 1st principal stress direction is parallel to the height direction, the size of the soilbag is unchanged when it is damaged, and the soil inside the bag and the soilbag reach the critical state of strength simultaneously When the soilbag is damaged, the size of the single-width tensile strength of the soilbag in each direction is equal, and the value of the soilbag is T (kN/m).

When the soilbag was in the critical damage state, the tension of the bag increased the effective stress of the soil inside the bag. A soil unit of tiny size is selected for the analysis, and the stress increments caused by the bag tension in the x, y and z directions are *σ*_1b_, *σ*_2b_, *σ*_3b_, respectively:

3 $$\left\{ \begin{gathered} \sigma_{{1{\text{b}}}} = \frac{2T}{B} + \frac{2T}{L} \hfill \\ \sigma_{{2{\text{b}}}} = \frac{2T}{H} + \frac{2T}{B} \hfill \\ \sigma_{{3{\text{b}}}} = \frac{2T}{H} + \frac{2T}{L} \hfill \\ \end{gathered} \right..$$

At this point, the principal stresses *σ*_1_, *σ*_2_ and *σ*_3_ of the soil in the bag are:

4 $$\left\{ \begin{gathered} \sigma_{1} = \sigma_{{1{\text{f}}}} + 2T(\frac{1}{B} + \frac{1}{L}) \hfill \\ \sigma_{2} = \sigma_{{2{\text{f}}}} + 2T(\frac{1}{H} + \frac{1}{B}) \hfill \\ \sigma_{3} = \sigma_{{3{\text{f}}}} + 2T(\frac{1}{H} + \frac{1}{L}) \hfill \\ \end{gathered} \right.,$$

According to the Mohr–Coulomb damage criterion, the critical strength of the soil in the bag is:

5 $$\sigma_{1} = K_{p} \sigma_{3} + 2c\sqrt[{}]{{K_{p} }},$$

6 $$\sigma_{{1{\text{f}}}} = \sigma_{{3{\text{f}}}} K_{{\text{p}}} + 2c\sqrt {K_{{\text{p}}} } + \left[ {2T\left( {\frac{1}{H} + \frac{1}{L}} \right)K_{{\text{p}}} - 2T\left( {\frac{1}{B} + \frac{1}{L}} \right) = \sigma_{{3{\text{f}}}} K_{{\text{p}}} + 2(c + c_{{\text{T}}} )\sqrt {K_{{\text{p}}} } } \right],$$

7 $$c_{{\text{T}}} = \frac{T}{{\sqrt {K_{{\text{p}}} } }}\left[ {\left( {\frac{1}{H} + \frac{1}{L}} \right)K_{{\text{p}}} - \left( {\frac{1}{B} + \frac{1}{L}} \right)} \right],$$

where:*c*, *c*_T_, *K*_p_ denote the cohesive force of the filling material, cohesion generated by soilbag wrapping, passive earth pressure coefficient of the filling material, respectively;

The load *F* of the soilbag at failure is predicted as follows:

8 $$F = \sigma_{{1{\text{f}}}} BL = \sigma_{{3{\text{f}}}} K_{{\text{p}}} BL + 2c\sqrt {K_{{\text{p}}} } BL + [2T(\frac{BL}{H} + B)K_{{\text{p}}} - 2T(L + B)]$$

#### Comparison of theoretical and test results

**Figure 24:**
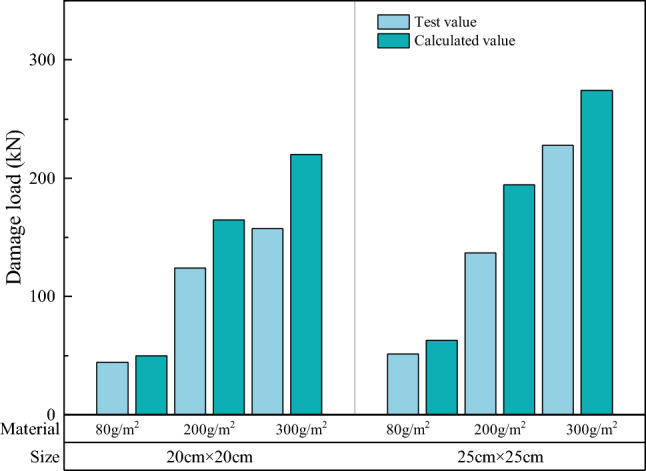
Comparison of theoretical and experimental values of surface damage loads.

**Figure 25:**
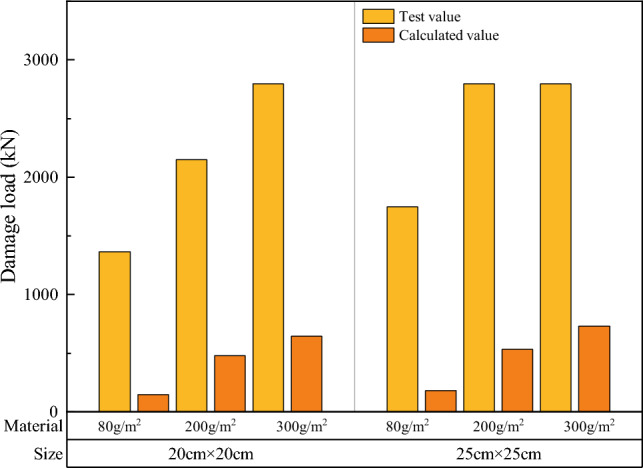
Comparison of theoretical and experimental values of overall damage loads.

The width and height of the specimen at the time of damage are substituted into Eq. (8), and compared with the measured results. Figure 24 presents a comparison between the theoretical and experimental values of the breaking load on the surface of a single soilbag, and Fig. 25 presents a comparison between the theoretical and experimental values of the overall breaking load of a single soilbag.

Figure 24 shows that the theoretical value of the surface damage of the single-layer soilbags was higher than the measured value, and the experimental value was approximately 78.6% of the theoretical value. Conversely, Fig. 25 demonstrates that the tested value of the overall damage load of a single-layer soilbag was significantly higher than the theoretical value, and the tested value was approximately 7.82 times the theoretical value.

**Figure 26:**
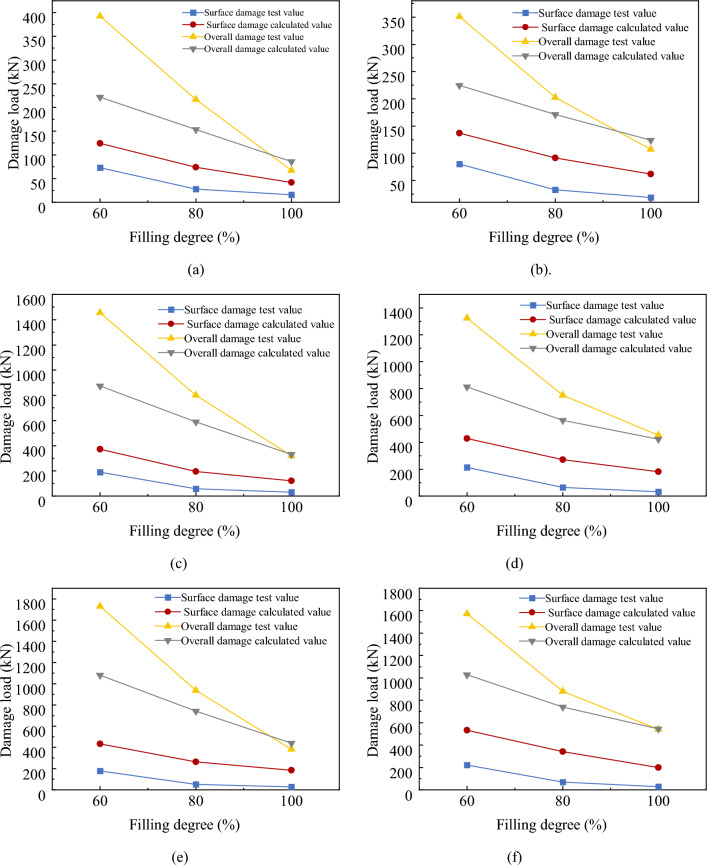
Measured and theoretical values of the destructive load of three-layer soilbags at different filling degrees: (**a**) 80 g/m^2^, 20 cm (**b**) 80 g/m2, 25 cm (**c**) 200 g/m^2^, 20 cm (**d**) 200 g/m^2^, 25 cm (**e**) 300 g/m^2^, 20 cm, and (**f**) 300 g/m^2^, 25 cm.

Figure 26 presents a comparison between the theoretical and measured values for the multi-layer soilbags.

As shown in Fig. 26, the measured values of the surface damage loads for the multi-layer soilbags were lower than the calculated theoretical values, and were approximately 50.2%, 28.1%, and 23.4% of the theoretical values at 60%, 80%, and 100% filling degrees, respectively. For the overall destructive load, the experimental values were significantly higher than the theoretical values at 60% infill, and these values gradually approached the theoretical values as the infill increased. The calculations showed that the overall damage loads of soilbags with 60%, 80%, and 100% filling degrees were approximately 1.626, 1.291, and 0.924 times the theoretical values, respectively.

The results of the above comparisons show that there is a significant difference between the damage loads calculated using the Matsuoka 3D force analysis model and the measured values. Based on a previous analysis of the influencing factors, the surface damage of soilbags is affected by the friction between the layers, and a relationship exists between the friction between the layers and the type of material, bag size, and degree of filling. Soil bags cause overall damage, wrapped soil causes a large degree of compression, and the role of the bag in the soil ranges from overall to lateral binding. Therefore, the original force analysis model is no longer applicable to the calculation of either surface or overall damage loads. To calculate the damage load of soilbags accurately, the original model can be revised, or a new force analysis model can be established by clarifying the damage and deformation characteristics and considering the influence of various factors.

## Conclusion

In this study, the geometric characteristics of soilbags with different degrees of filling were analyzed via geometric shape tests of water-filled sealed bags. Further uniaxial compression tests on soilbags filled with graphite tailings of different layers, soilbag materials, filling degrees, and sizes, the load-deformation relationship and damage characteristics during compression were obtained. The effect of each factor on the damage load was analyzed, and the damage load was compared with the results of the Matsuoka theory calculations. The conclusions are as follows:

1. The volume of the soilbag expressed in the cubic form was approximately 0.773 times the actual volume, and the heights of the soilbags with different fill levels derived from this relationship deviated from the measured heights by less than 10%. The method’s judgment of the initial shape of the soilbags for each degree of filling can be used as an important basis for soilbag strength calculations and stacking applications.
2. Damage to graphite tailing-filled soilbags can be defined as surface damage and overall damage. The compression deformation was approximately 14.6% of the initial height in the case of surface damage to single-layer soilbags, and approximately 45.1% of the initial height in the case of overall damage. For the three-layer soilbags with filling degrees of 100%, 80%, and 60%, the compression deformations when surface damage occurred were 6.476%, 10.778%, and 20.245% of the initial height, respectively, and the corresponding compression deformations when overall damage occurred were 39.766%, 43.674%, and 45.43% of the initial height.
3. The compressive properties of the soilbags filled with graphite tailings were affected by the number of soilbag layers, material, degree of filling, and soilbag size. Multi-layer soilbags can better reflect the actual force situation than single-layer soilbags. The surface damage load increased with decreasing fill and decreased with decreasing soilbag size. The surface damage load was greater for bags with high tensile capacity and low interlayer friction. The overall damage load increased with an increase in the tensile strength of the soilbag material, decreased with an increase in the degree of filling, and was greater for large-sized soilbags at high degrees of filling.
4. For soilbags filled with graphite tailings in three layers at 60%, 80%, and 100% filling, the experimental values of the surface damage load were approximately 50.2%, 28.1%, and 23.4% of the theoretical values, respectively, while those of the overall damage load were approximately 1.626, 1.291, and 0.924 times the theoretical values, respectively. A comparison of these results indicates that the original force analysis model cannot accurately calculate the damage load of soilbags filled with graphite tailings; thus, the original model should be revised or a new force analysis model should be established.

## Data Availability

The datasets used in this investigation are accessible for review upon request from the paper’s corresponding author.
